# Reduction in the presence of cryoglobulins over time in the hemodialysis treatment

**DOI:** 10.1080/0886022X.2017.1349676

**Published:** 2017-07-25

**Authors:** Tainá Mosca, Simone Sanches Arcuri, Luiz Antônio Miorin, Andrelita de Oliveira Cristiano, Yvoty Alves dos Santos Sens, Wilma Carvalho Neves Forte

**Affiliations:** a Department of Pathological Sciences, Santa Casa de São Paulo School of Medical Sciences, São Paulo, SP, Brazil;; b Department of Medicine, Santa Casa de São Paulo School of Medical Sciences, São Paulo, SP, Brazil;; c Department of Postgraduate, Santa Casa de São Paulo School of Medical Sciences, São Paulo, SP, Brazil

**Keywords:** Hemodialysis, cryoglobulins, renal failure, immunocomplexes, cryoprecipitate

## Abstract

**Background:** The presence of cryoglobulins in patients with chronic kidney disease (CKD) on hemodialysis is well described. However, the generation of cryoglobulins during the dialysis treatment has yet to be established. The aim of the present study was to determine the presence of serum cryoglobulins over time in the dialysis treatment in patients with CKD not infected with hepatitis C virus (HCV).

**Method:** Peripheral blood samples were collected at the beginning of dialysis treatment and at 30, 60, 90 and 120 days afterwards. Cryoglobulins were defined by the presence of immunocomplexes that precipitated *in vitro* with exposure to cold and resolubilized when rewarmed. The components of the cryoprecipitate were analyzed by radial immunodiffusion.

**Results:** In this study, 14 patients were included: 11 male and three female, aged 28–88 years, with mean time on hemodialysis of 57 ± 36 days at baseline. The presence of cryoglobulin, constituted by IgM, IgA, IgG and the C3 and C4 components of the complement, was observed in the serum of all patients at the beginning of hemodialysis. Sequence analyses showed that the amount of cryoprecipitate decreased during the dialysis treatment.

**Conclusion:** There was a high prevalence of mixed cryoglobulins in CKD patients at the beginning of hemodialysis, and the amount of cryoprecipitate decreased during the treatment.

## Introduction

Chronic kidney disease (CKD) is defined as a decrease in renal function and in the glomerular filtration rate (GFR), for three months or more. The values of GFR define the progression stages of the disease, with dialysis treatment being required for extra-renal clearance in most advanced stage [1,2].

The main causes of CKD are arterial hypertension, glomerulonephritis and diabetes mellitus, and it may also be due to polycystic kidney disease, systemic lupus erythematosus, frequent urinary tract infections and other infections such as those caused by the hepatitis C virus (HCV) [1,3].

The role of inflammation and the immune system in the pathogenesis of CKD has been considered [4,5]. Renal lesion can be triggered by various immunologic mechanisms, such as specific antibodies that bind to glomerular structures, local activation of the complement system, local activation and migration of leukocytes and circulating immunocomplex deposition [3,6].

Cryoglobulins are circulating immunocomplexes consisting of antibodies, antigens and complement, usually subsequent to infections or autoimmune diseases. Circulating immunocomplexes precipitate when exposure to low temperatures and solubilize when heated. Cryoglobulins are classified as types I, II and III. Type I (monoclonal) consist of a single immunoglobulin isotype, and type II and III cryoglobulins (mixed) are formed by more than one immunoglobulin class (IgG, IgM, or IgA) [4]. The greater number of antigen molecules in relation to those of antibodies leads to the formation of soluble immunocomplexes in the bloodstream, which may cause type-III hypersensitivity or immunocomplex reactions, resulting in tissue lysis [4,7].

In CKD, cryoglobulin formation may be depend on the continued presence of antigens, as in the case of HCV infection, or the production of autoantibodies that are determinants of glomerulonephritis. It may also be associated with diseases such as HIV infection, Sjögren’s Syndrome, polyarthritis and B-cell lymphoma [4,8].

The presence of cryoglobulins in CKD has been demonstrated in patients undergoing dialysis. The major of these patients are HCV positive [9]. However, in a previous study, we conclude that the presence of cryoglobulins in CKD is not always associated with HCV presence: cryoglobulins were observed in 83% of 54 patients on chronic hemodialysis, and among them, only 25 were infected with HCV [13].

To date, the evolution of cryoglobulins during the dialysis treatment of patients with CKD, HCV negative has not been established yet. Thus, considering the importance of CKD and the possible role of cryoglobulins on the disease aggravation, studies concerned cryoglobulins are important. Therefore, the aim of the present study was to assess the presence of serum cryoglobulins over time in the dialysis treatment in patients with chronic kidney disease.

## Patients and methods

The project was approved by the institution’s Research Ethics Committee, under number 527,996. All study participants signed an Informed Consent Form.

### Subjects

Patients of both genders, aged above 21 years, were selected from the Nephrology Sector at a tertiary hospital located in downtown area of São Paulo city. The study of cryoprecipitates was conducted in the Immunology Laboratory at the same institution.

Inclusion criteria were patients starting dialysis treatment (less than four months) with no clinical or laboratory diagnosis of autoimmune disease, infectious processes, transfusions or immunizations in the past three months. Exclusion criteria were patients infected with the hepatitis C virus (HCV), smokers or ex-smokers, obese, renal transplant recipients, patients receiving immunosuppressive drugs, having undergone recent surgeries, with allergic processes (except rhinitis) and patients with immunodeficiencies or other associated diseases.

All patients underwent hemodialysis three times weekly, using polysulfone F80 capillary dialyzers, 800 mL/min flow with ultrapure water. Time on hemodialysis, serum creatinine and urea levels were analyzed at start of treatment.

### Detection of cryoglobulins

Blood samples were obtained from patients starting hemodialysis treatment and 30, 60, 90 and 120 days afterwards, according to the inclusion and exclusion criteria. The presence of serum cryoglobulins was determined by the formation of cryoprecipitate [14,15]. Briefly: 10 mL blood samples were collected in a warmed (37 °C) dry tubes and were allowed to clot at 37 °C. After centrifugation (330 *g*, 10 min, 25 °C), 4 mL serum was separated and stored at 4 °C for 21 days. The formed cryoprecipitates were classified according to size: one cross stands for the smallest quantity visualized and four crosses, the largest. The cryoprecipitates were then centrifuged (1000 *g*, 10 min, 4 °C), washed 10 times in saline and stored at −4 °C for the quantification of their components. The quantification of the cryoprecipitates components was determined by radial immunodiffusion plates for IgM, IgA, IgG and the C3 and C4 components of the complement (NOR Partigen, Siemens Healthcare Diagnostics, Marburg, Germany). Parallel heating tests (37 °C) were performed on the cryoprecipitate, which precipitated at low temperatures and became soluble when heated.

### Statistical analysis

The comparison of the quantification of IgM, IgA, IgG, C3 and C4 results from initial and final measurements was analyzed by the nonparametric Wilcoxon test, after using the Kolmogorov–Smirnov normality test. *p* values <.05 were considered significant.

## Results

In this study, 14 patients were included, 11 male and three female, aged 28–88 years (mean age of 53 ± 15 years).

At the beginning of the study, patients were undergoing hemodialysis treatment for a period of time that ranged from 7 to 124 days (with an average of 57 ± 36 days). During follow-up, two patients received kidney transplantation (patients 1 and 14), one died (patient 2), and two were transferred to another hospital (patients 4 and 8). Table 1 shows the quantification by observational criterion (depicted as crosses) of the size of cryoprecipitates formed in the patients’ serum, during hemodialysis treatment.

**Table 1.** Size of cryoprecipitates: Quantification, by observational criteria, of the presence of cryoprecipitate in the serum of patients with chronic kidney disease on hemodialysis (HD).

Patient	Time on HD (days)	1st Sampling	30 days	60 days	90 days	120 days
1	7	+ + + +	+ + + +	+ + +	+ +	Transplanted
2	15	+ + + +	+ + +	+	Died	- - - -
3	25	+ +	+ +	+	+	+
4	30	+ + + +	+ +	+ +	Transferred	- - - -
5	30	+ + +	+ +	+ +	+ + +	+
6	41	+ +	+ +	+ +	+ +	+ +
7	55	+ + + +	+	+	+	+
8	54	+	+	Negative	Transferred	- - - -
9	60	+ + + +	+	+	+	+
10	67	+ + +	+ +	+ + +	+ +	+ + +
11	80	+	Negative	Negative	+	Negative
12	87	+	- - - -	+	+	- - - -
13	120	+	+	+	+	+
14	124	+	Negative	+	Negative	Transplanted

Data obtained from a first sampling and 30, 60, 90 and 120 days afterwards. Qualification of the cryoprecipitate: + (smaller in size); ++++ (larger in size); - - - -: no material for analysis; negative: without cryoprecipitate formation.

Table 2 shows the quantitation of IgM, IgA, IgG, C3 and C4 in the cryoprecipitate formed in the sera of patients in the initial and final sample measurements with the presence of cryoprecipitate. In the comparison of these values, according to the Wilcoxon test (Table 3), the concentrations of IgM, IgA, IgG, C3 and C4 in the initial measurement were significantly higher than those in the final measurement (*p* < .05).

**Table 2.** Quantification of cryoprecipitate: Concentration levels (mg/dL) of components formed in the serum of patients with chronic kidney disease on dialysis: data from initial and final sample measurements.

	Initial					Final				
Patient	IgM	IgA	IgG	C3	C4	IgM	IgA	IgG	C3	C4
1	19.1	129.8	662.6	79.3	26.5	3.4	83.5	235.3	51.8	10.8
2	122.3	509.9	1810.9	143.1	63.1	3.4	42.1	86.5	9.5	2.1
3	3.5	12.5	121.5	9.5	2.1	3.4	12.5	86.5	9.5	2.1
4	41.0	58.1	435.3	31.8	10.8	32.0	34.4	232.0	27.1	2.1
5	3.6	92.4	310.5	41.5	14.9	3.4	12.5	121.5	9.5	3.7
6	3.5	12.5	194.2	9.5	2.1	3.4	12.5	121.5	9.5	2.1
7	23.4	83.5	392.6	36.6	9.8	3.4	12.5	86.5	9.5	2.1
8	3.4	12.5	86.5	9.5	2.1	3.4	12.5	86.5	9.5	2.1
9	19.1	129.8	662.6	79.3	26.5	3.4	83.5	235.3	51.8	10.8
10	19.1	74.8	310.5	27.1	5.3	19.1	120.2	87.5	36.6	5.3
11	3.4	12.5	86.5	9.5	2.1	3.4	12.5	86.5	9.5	2.1
12	3.4	12.5	86.5	9.5	2.1	3.4	12.5	86.5	9.5	2.1
13	3.4	42.1	232.0	9.5	2.1	3.4	12.5	86.5	9.5	2.1
14	3.4	12.5	86.5	9.5	2.1	3.4	12.5	86.5	9.5	2.1

**Table 3.** Statistical analysis: Comparison of the results from the quantification (mg/dL) of the components of the cryoprecipitate formed in the beginning and at the end of the study.

	IgM	IgA	IgG	C3	C4
Mean - Initial ± SD	19.4 ± 31.8	85.4 ± 129.7	391.3 ± 454.6	36.1 ± 39.5	12.3 ± 17.0
Mean - Final ± SD	6.6 ± 8.4	34.0 ± 35.7	123.2 ± 61.4	18.7 ± 16.2	3.7 ± 3.1
95% CI - Initial	1.07–37.73	10.48–160.29	128.88–653.79	13.29–58.88	2.42–22.10
95% CI - Final	1.69–11.43	13.41–54.62	87.76–158.68	9.36–28.11	1.87–5.54
*p* value	.012	.035	.005	.042	.027

Mean, ± standard deviation (SD) and 95% confidence interval (CI) of concentration values results from initial and final measurements. *p* values for the comparison of initial and final parameters, according to the Wilcoxon test.

## Discussion

The results showed that all patients studied had cryoglobulins in peripheral blood. The cryoglobulin found was mixed, consisting of IgM, IgA, IgG and the C3 and C4 components of the complement. The results also showed that cryoglobulinemia decreased during the hemodialysis treatment.

In the literature, most studies on CKD and cryoglobulins were performed with patients infected with HCV and on chronic dialysis [5,9–12], it has been shown the presence of mixed cryoglobulins in their serum [9,13,16]. Mascia et al. [17], in a review article, report that 90% of cases cryoglobulinemia are associated with HCV infection. According to the authors, in the case of individuals HCV negative, the presence of cryoglobulins is related with other infections, such as HIV, or with autoimmune diseases and neoplastic disorders. Other studies suggest that the formation of cryoglobulins, in patients with CKD and HCV negative, is the result of some disorders in immune system caused by hemodialysis [11,13,17,18]. It has already been reported that hemodialysis patients have decreased phagocytic activity, cellular dysfunction of NK cell, B and T lymphocytes, increased production of inflammatory cytokines and activation of the complement system [6,19–22]. All these immunological disorders can contribute to the clearance of immunocomplexes is not efficient, possibly resulting in cryoglobulins formation.

The results obtained in this study suggest that the dialysis aids in reducing cryoglobulin, because the concentration of cryoprecipitate decreases during the treatment. Indeed, patients on hemodialysis for lesser periods of time exhibited a larger quantity of cryoprecipitate (larger in size and greater levels of IgM, IgA, IgG, C3, C4) as compared to patients receiving dialysis treatment for longer periods. Thus, individuals with cryoprecipitate quantified as ranging from three to four to crosses had initiated hemodialysis treatment more recently than those quantified with one cross (Table 1).

To date, no studies are available in the literature on the presence of cryoglobulins at the beginning of dialysis treatment in patients with CKD and negative for HCV, or studies following the formation of cryoglobulins during treatment. Therefore, this is one of the first studies evaluating the presence of cryoglobulins over time in the hemodialysis treatment in patients with CKD and not infected with HCV.

In the present study, the treatment used was the hemodialysis using polysulfone capillary and ultra-pure water. According to the literature, this treatment is the option that causes the lowest number of immune and inflammatory reactions when compared to other devices using cellulose membrane [19,23]. Accordingly, our results suggest that there are other causes for cryoglobulin formation than dialysis. Those causes could precede the hemodialysis treatment.

A possible trigger for the cryoglobulin formation could be the patients’ uremic state. In uremia, there are several toxins such as urea, lipids and peptides that may have pro-inflammatory effects, that can activate the immune system [6,24]. It has previously been reported that uremic subjects have IgM-anticardiolipin antibodies in their serum [6,9], which could cause the immunocomplexes formation. Furthermore, there are studies showing that, in uremic individuals, monocytes and neutrophils have a decreased phagocytic activity. Monocytes and neutrophils are important for the clearance of immunocomplexes, and dysfunctions in their responses may contribute to the persistence of circulating immunocomplexes [6].

Thus, this paper support the importance and need to assess the presence of cryoglobulin early in diagnosis of renal failure, at the time a conservative treatment is established, since cryoglobulinemia can be one of the causes of the progression of kidney damage associated with CKD.

In conclusion, this study found that all studied patients with chronic kidney disease on hemodialysis, not infected with HCV, presented serum crioglobulin at the beginning of dialysis and that the amount of cryoprecipitate declined during this treatment.
